# Tempol Induces Oxidative Stress, ER Stress and Apoptosis via MAPK/Akt/mTOR Pathway Suppression in HT29 (Colon) and CRL-1739 (Gastric) Cancer Cell Lines

**DOI:** 10.3390/cimb47070574

**Published:** 2025-07-21

**Authors:** Gorkem Ozdemir, Halil Mahir Kaplan

**Affiliations:** 1 Department of Gastroenterological Surgery, Adana City Training and Research Hospital, 01230 Adana, Turkey; 2 Department of Pharmacology, Faculty of Medicine, Çukurova University, 01330 Adana, Turkey; mkaplan@cu.edu.tr

**Keywords:** tempol, apoptosis, *Akt/mTOR* pathway, colon cancer, CRL-1739, HT29, gastric cancer, *MAPK* pathway

## Abstract

Tempol is a synthetic antioxidant that shows promise in preclinical cancer studies by inhibiting growth and inducing apoptosis. Given that the Mitogen-Activated Protein Kinase (MAPK) and Protein Kinase B/Mammalian Target of Rapamycin (Akt/mTOR) signaling pathways are frequently dysregulated in gastric and colon cancers and contribute to their progression, we investigated Tempol’s anti-cancer potential in HT29 (colon) and CRL-1739 (gastric) cancer cells. Cells were treated with 2 mM Tempol for 48 h, with untreated cells as controls. We evaluated apoptosis (Bax, cleaved caspase-3, and Bcl-2), key signaling pathway activity (*p*-ERK, *p*-JNK, *p*-AKT, and *p*-mTOR), and levels of stress- and apoptosis-related proteins (WEE1, GADD153, GRP78, and AIF). Tempol significantly increased pro-apoptotic Bax and cleaved caspase-3 (*p* < 0.0001) and decreased anti-apoptotic Bcl-2 (*p* < 0.0001) in both cell lines. Furthermore, Tempol markedly reduced the activity of *p*-ERK, *p*-JNK, *p*-AKT, and *p*-mTOR (*p* < 0.0001) and significantly increased the protein levels of WEE1, GADD153, GRP78, and AIF (*p* < 0.0001). Tempol treatment also led to a significant increase in total oxidant status and a decrease in total antioxidant status. In conclusion, our findings suggest that Tempol exhibits its anti-cancer activity through multiple interconnected mechanisms, primarily inducing apoptosis and oxidative stress, while concurrently suppressing pro-survival signaling pathways. These results highlight Tempol’s potential as a therapeutic agent for gastric and colon cancers.

## 1. Introduction

Introduced in 1960, Tempol is a highly effective catalyst widely used in chemical synthesis and oxidation. Beyond its catalytic applications, research has explored Tempol’s potential in treating a diverse range of conditions linked to oxidative stress, including inflammation, neurological disorders, cardiovascular issues, eye damage, skin injuries, fibrocystic diseases, cancer prevention, respiratory infections, hair loss, and cerebral malaria [1].

Tempol’s biological effects stem from its unique redox-cycling capabilities, allowing it to act as both an oxidant and an antioxidant depending on the specific cellular context and concentration. This dual nature is crucial; for instance, in lung and prostate cancer cells, Tempol concentrations ranging from 0.5 to 4 mM can either increase or decrease reactive oxygen species (ROS) levels depending on the cell type and experimental conditions. Notably, 1 mM Tempol has been shown to significantly inhibit cell growth and induce cell death, which correlates with elevated superoxide levels and glutathione depletion [2,3]. Its modulation of nitric oxide metabolites and antioxidant status also varies by tissue, indicating complex, tissue-dependent effects [4]. This dynamic interaction suggests that Tempol’s efficacy is highly context-dependent.

Moreover, Tempol has demonstrated promising anti-cancer potential in preclinical studies. It has been observed to inhibit the growth of lung cancer cells by inducing apoptosis, again associated with increased ROS levels and glutathione depletion [2]. Cancer cells commonly exhibit elevated levels of ROS, which paradoxically can promote tumor growth and survival [3,5]. Nevertheless, strategic alteration of ROS levels offers a pathway to disrupt cancer cell proliferation, induce apoptosis, and inhibit angiogenesis [6]. Interestingly, Tempol also acts as a radiation protector, selectively reducing radiation-induced cytotoxicity in normal cells while preserving the sensitivity of tumor cells to radiation [7,8]. Despite these encouraging findings, Tempol remains primarily an experimental agent and has not been widely adopted in clinical practice [9,10,11]. Crucially, there are limited data in the existing literature on the topic regarding its specific effects on gastric and colon cancer cells, which represents a significant research gap.

The Mitogen-Activated Protein Kinase (MAPK) and Protein Kinase B/Mammalian Target of Rapamycin (Akt/mTOR) pathways are crucial signaling networks in fundamental cellular processes like growth, proliferation, and survival. Their clinical relevance in gastric and colon cancers stems from their frequent dysregulation, often driven by genetic alterations (e.g., *KRAS*, *BRAF*, *PIK3CA*, *PTEN*, and *mTOR*), which leads to constitutive activation and fosters uncontrolled cancer cell growth and survival. Consequently, these pathways are attractive therapeutic targets in gastric and colon cancers. While inhibitors targeting components of the MAPK pathway (e.g., *MEK* and *BRAF*) have shown some clinical benefit in colorectal cancers, their efficacy as single agents in gastric cancer is limited. Similarly, PI3K, Akt, and mTOR inhibitors are being evaluated in clinical trials for both gastric and colon cancers [12,13]. Despite some promise, challenges like toxicity and resistance persist.

Therefore, the aim of this study is to determine if Tempol exerts anti-cancer activity in HT29 colon and CRL-1739 gastric cancer cells through the induction of apoptosis and oxidative stress and the suppression of MAPK and Akt/mTOR signaling pathways.

Specifically, this study aims to elucidate the mechanisms by which Tempol exerts its anti-cancer effects by examining its impact on the following parameters:

Apoptosis: (assessing the levels of pro-apoptotic (Bax and cleaved caspase-3) and anti-apoptotic (Bcl-2) proteins);

Key signaling pathways: evaluating the activity (phosphorylation levels) of crucial molecules involved in cell growth and survival, namely ERK, JNK, AKT, and mTOR;

Cellular stress responses and apoptosis-related proteins: analyzing the changes in the levels of proteins like WEE1, GADD153, GRP78, and AIF, which are often involved in cellular stress responses and apoptosis.

## 2. Materials and Methods

This study was conducted and completed in the Department of Pharmacology, Faculty of Medicine, Çukurova University, between June 2024 and May 2025.

### 2.1. Chemicals

McCoy’s 5A Medium, RIPA buffer, fetal bovine serum, PBS, NaCl, TritonX-100, EGTA, dithiothreitol, NaF, Tris–HCl, and Na3VO4 were obtained from Sigma-Aldrich, Inc. (St. Louis, MO, USA). In addition, the Bradford dye reagent was purchased from Bio-Rad Laboratories, Inc. (Hercules, CA, USA). Tempol was obtained from Sigma-Aldrich Co. Total Antioxidant Status (TAS) and Total Oxidant Status (TOS) assays were purchased from Rel Assay Diagnostics Inc. (Gaziantep, Turkey).

### 2.2. Cell Line and Culture Conditions

The human colon cancer cell line HT29 was obtained from the American Type Culture Collection (ATCC) (Manassas, VA, USA). Cells were cultured in modified McCoy’s 5A Medium supplemented with 10% fetal bovine serum. The cells were incubated in a humidified atmosphere at 37 °C with 5% CO_2_ [14].

Human gastric adenocarcinoma cells, CRL-1739 (AGS), were also obtained from ATCC. These cells were maintained in F-12 medium (Gibco, Waltham, MA, USA) supplemented with streptomycin (100 μg/mL), penicillin (100 U/mL) (Sigma, St. Louis, MO, USA), and 10% fetal bovine serum (FBS) (Gibco, Waltham, MA, USA). The cells were incubated at 37 °C in a humidified atmosphere containing 5% CO_2_.

Cells were maintained through regular passaging, and experiments were performed using cells within the passage range of 3 to 15.

### 2.3. Cell Homogenization

Cells (5 × 10^4^ cells/cm^2^) were exposed to 2 mM Tempol for 48 h. Following this, cells were washed with PBS and lysed in RIPA buffer (containing 150 mM NaCl, 0.5% Triton X-100, 20 mM EGTA, 1 mM dithiothreitol, 25 mM NaF, 50 mM Tris-HCl [pH 7.4], and 1 mM Na_3_VO_4_) for 15 min on ice. The lysate was then centrifuged at 15,000 rpm for 20 min, and the supernatant was collected while the pellet was discarded.

### 2.4. Total Protein Quantification

Protein concentration in cell homogenates was determined using the Bradford method. A standard curve was generated using bovine serum albumin (BSA) at concentrations ranging from 1 to 10 μg/mL. A total of 10 μL of each sample was diluted to 100 μL with distilled water. Subsequently, 1 mL of Bradford reagent was added to both standards and samples, followed by vortexing. Absorbance at 595 nm was measured manually using a spectrophotometer. Protein concentration in μg/μL was determined by interpolating sample absorbance values onto the standard curve generated using Prism software version 9.5.1. Protein quantification (using the Bradford method) was performed six times independently.

### 2.5. ELISA (Enzyme-Linked Immunosorbent Assay) Test

Direct ELISA assays were performed to determine the proteins levels and activity of cleaved caspase-3, Bax, Bcl-2, p-JNK, p-ERK, p-AKT, p-mTOR, WEE1, GADD153, GRP78, and AIF proteins. Each ELISA test was repeated 6 times independently.

R&D Systems ELISA Kits (R&D Systems, Inc., Minneapolis, MN, USA) were used.

### 2.6. Treatment Groups

A 48 h treatment allows detection of both immediate cytotoxic effects and early cellular responses such as apoptosis induction, cell cycle arrest, or changes in signaling pathways. Longer treatments (e.g., 7 days) can introduce confounding factors such as cell adaptation, recovery, or the emergence of resistant subpopulations. A 48 h window limits these effects. Many anti-cancer drugs require sustained exposure to exert effects, and 48 h simulates a clinically relevant window of drug action without prolonged continuous exposure that may not mimic patient pharmacokinetics. It is a manageable timeframe for routine assays like viability, apoptosis, and molecular analyses, balancing experimental throughput and biological relevance [15,16].

Based on the data in this study, HT29 and CRL-1739 cells were treated with 2 mM Tempol for 48 h. Untreated HT29 and CRL-1739 cells served as controls.

### 2.7. Statistical Analysis

Statistical analyses were performed using GraphPad Prism 10.0 (GraphPad Software, San Diego, CA, USA). Data were analyzed using unpaired Student’s *t*-tests to compare parameters between control and Tempol-treated groups. Data are presented as means ± standard deviation (SD). Statistical significance was defined as *p* < 0.05.

## 3. Results

### 3.1. Tempol Promotes Apoptosis of HT29 and CRL1739 Cells by Modulating Pro-Apoptotic and Anti-Apoptotic Proteins

As presented in Table 1, Tempol treatment resulted in a highly significant (*p* < 0.0001) increase in Bax and cleaved caspase-3 protein levels compared to the control groups in both gastric (CRL1739) and colon (HT29) cancer cells. Conversely, Tempol treatment significantly (*p* < 0.0001) decreased Bcl-2 levels in both cell lines compared to controls.

### 3.2. Tempol Reduces the Phosphorylation Levels of Key Signaling Molecules (ERK, JNK, AKT, and mTOR) Involved in Cell Growth and Survival

As presented in Table 2, Tempol treatment resulted in a significant reduction in the levels of phosphorylated ERK, JNK, AKT, and mTOR in both gastric cancer cells (CRL-1739-TPL) and colon cancer cells (CC-TPL) when compared to their respective control groups. All reductions were statistically significant (*p* < 0.0001).

### 3.3. Tempol Increases the Levels of Cellular Stress- and Apoptosis-Related Proteins (WEE1, GADD153, GRP78, and AIF)

As presented in Table 3 Tempol treatment significantly increased the protein levels of *GRP78*, *AIF*, *GADD153*, and *WEE1* in both gastric and colon cancer cells compared to the control groups (*p* < 0.0001 for all).

### 3.4. Tempol Induces Oxidative Stress and Depletes Antioxidant Capacity in Cancer Cells

As presented in Table 4, Tempol administration resulted in a highly significant increase in Total Oxidant Status (TOS) and a concomitant significant decrease in Total Antioxidant Status (TAS) in both HT29 and CRL-1739 cells compared to untreated controls (*p* < 0.0001).

## 4. Discussion

Tempol has demonstrated potential in cancer therapy, including the ability to inhibit the growth of lung cancer. This inhibition involves inducing apoptosis in both cancer and normal cells, which is associated with increased levels of ROS and glutathione depletion [2]. However, the literature on the topic contains limited data concerning its effects on gastric and colon cancer cells. Therefore, our study specifically focused on investigating the anti-cancer effects of Tempol on these cell types. The MAPK (Raf-MEK-ERK) and Akt/mTOR pathways are crucial signaling networks that govern cell differentiation, proliferation, and survival. Their frequent dysregulation drives uncontrolled growth and apoptosis resistance in cancer. Consequently, inhibiting these pathways is a promising cancer therapy strategy, which is why this study investigated Tempol’s impact on these key pathways and its apoptotic effects in colon and gastric cancer cells.

Our findings reveal Tempol’s pro-oxidant potential at millimolar concentrations by not only inducing oxidative stress, but also reducing the cellular antioxidant capacity in gastric and colon cancer cells. Tempol, at the tested concentration, acts as a pro-oxidant and induces severe oxidative stress. This disruption of redox homeostasis and oxidative stress seems to act as a primary trigger, directly leading to cellular dysfunction, activation of ER stress pathways (GADD153 and GRP78 upregulation) and concomitant suppression of major pro-survival signaling pathways (MAPK and Akt/mTOR). The increase in ROS and the cells’ inability to neutralize them activate pro-apoptotic pathways, evidenced by increased Bax and cleaved caspase-3 and decreased Bcl-2. This oxidative stress also likely contributes to the inhibition of key pro-survival signaling pathways (ERK, JNK, AKT, and mTOR), as these pathways are sensitive to changes in the cellular redox environment. The upregulation of stress-related proteins like GRP78, GADD153, and AIF further corroborates the induction of severe cellular stress, including ER stress.

The MAPK/ERK signaling cascade initiates when extracellular factors bind to receptors like receptor tyrosine kinases (RTKs), activating RAS proteins [17]. This pathway is frequently altered in cancers, with RTK-RAS pathway alterations occurring in approximately 37% of gastric cancers and *BRAF* mutations in up to 11% [18,19]. Activated RAS proteins then engage RAF kinases, which sequentially activate MEK1/2 and subsequently ERK1/2 [20,21,22]. As key regulators of proliferation, differentiation, and survival, elevated *p*-ERK1/2 levels correlate with poor gastric cancer survival [23,24]. In gastric cancer, this cascade further modulates cell motility, adhesion, and invasion [25,26].

The PI3K/AKT/mTOR pathway plays a vital role in the proliferation and survival of gastric cancer cells [27,28]. This signaling route facilitates the progression of gastric cancer by impeding apoptosis and fostering drug resistance, metastasis, and angiogenesis [29]. Alterations within the PI3K/AKT/mTOR pathway are critical in the development of resistance to both HER2-targeted therapies and chemotherapeutic drugs, not only in gastric cancer but also in other solid tumors [30,31]. This pathway is frequently dysregulated in gastric cancer [32,33]. Increased levels of AKT and its phosphorylated form (*p*-AKT) have been detected in over 74% of gastric cancer cases [34].

The significant increase in WEE1 suggests that Tempol might induce cell cycle arrest (likely at the G2/M checkpoint). This could be an attempt by the cell to repair damage (e.g., DNA damage induced by oxidative stress) before entering mitosis. If the damage is too extensive or the repair is unsuccessful, this arrest can lead to mitotic catastrophe and cell death. The significant increase in AIF (apoptosis-inducing factor) suggests a potential involvement of caspase-independent cell death pathways, or at least a pathway where AIF translocates from mitochondria to the nucleus to promote chromatin condensation and DNA fragmentation, even if caspases are also activated. This further implicates mitochondrial damage as part of Tempol’s mechanism. While our study focused on apoptosis, the marked increase in oxidative stress and ER stress markers warrants further investigation into whether or not Tempol also induces other forms of programmed cell death, such as autophagy or necroptosis, especially if apoptosis is inhibited or incomplete.

The JNK MAPK subgroup comprises three genes: MAPK8 (JNK1), MAPK9 (JNK2), and MAPK10 (JNK3) [35]. Activation of JNK can result in proliferation, apoptosis, or cellular transformation [36]. Notably, JNKs are capable of interacting with other MAPK pathways; for instance, JNK subtypes can activate p38-MAPK, and certain upstream regulators are shared between them. Research involving JNK1 knockout mice demonstrated a reduction in gastric carcinogenesis induced by N-methyl-N-nitrosourea [37]. Consequently, JNK1 appears to play a role in both the initiation and progression of tumors, positioning it as a potentially valuable target for gastric cancer prevention. Furthermore, p38-MAPK is selectively activated by upstream MAPK kinases.

Combined targeting of the AKT/mTOR and ERK pathways synergistically suppresses tumor growth, underscoring their cooperative pro-tumorigenic roles [12]. This approach has shown particular promise in models of prostate and breast cancers, where combination treatments with mTOR and MEK inhibitors were more effective than single agents, including against hormone-refractory prostate cancer [12]. Similarly, combined AKT and mTOR inhibition yielded synergistic anti-proliferative effects in colorectal cancer cell lines and patient-derived spheroids [38]. Our study aligns with these findings, demonstrating that Tempol also targets both AKT/mTOR and ERK proteins, suggesting its potential as a multifaceted anti-cancer agent.

Our study also shows that Tempol treatment significantly increased the protein levels of WEE1, GADD153, GRP78, and AIF. The upregulation of WEE1, a key cell cycle regulator, suggests Tempol induces G2/M cell cycle arrest to allow for DNA repair [39]. Similarly, elevated GADD153 indicates ER stress, which can trigger apoptosis if unresolved [40]. The increase in GRP78 points to activation of the unfolded protein response (UPR), a cellular mechanism to restore ER homeostasis [41]. While GRP78 can promote cancer progression by interacting with various proteins, including anti-apoptotic survivin in colon cancer cells [42], its concurrent increase with GADD153 suggests Tempol’s ability to drive ER stress toward pro-apoptotic outcomes. Of particular significance, the increased level of AIF suggests the initiation of apoptosis, potentially involving caspase-independent pathways. This combined upregulation of stress- and apoptosis-related proteins indicates Tempol activates multiple pathways leading to programmed cell death and reduced cancer cell survival.

AIF’s increased level underscores the multifaceted nature of Tempol’s action. Tempol triggers apoptosis by activating caspases, a key pathway in programmed cell death. This supports its potential as a chemotherapeutic agent. While promising in preclinical studies, further investigation is crucial for clinical translation. Rigorous clinical trials are needed to establish safe and effective dosing strategies. Studies are required to determine optimal administration routes and understand how Tempol is processed within the body.

This study, while providing promising in vitro evidence for Tempol’s anti-cancer effects, has several limitations. The most significant limitation is the absence of in vivo data. The cells were exposed to a single concentration (2 mM) of Tempol for a single duration (48 h). This limits the understanding of the dose–response relationship and the time-dependent effects of Tempol. The potential off-target effects of Tempol are another limitation. One main limitation of our study is that we only used one method, called ELISA, to look at protein levels and cell death (apoptosis). To be absolutely sure about our findings, it would have been better to use more ways to check these things. For example, to really confirm cell death, we should have used Western blotting, and also flow cytometry or other color tests. Similarly, confirming changes in protein levels with Western blotting and seeing how cell division changes with flow cytometry would have made our conclusions much stronger. We also acknowledge that our study did not evaluate Tempol’s effect on healthy proliferating cells. This limits our understanding of its specificity towards cancer cells and its potential therapeutic window or side effects. Furthermore, investigating the effects of Tempol in 3D culture conditions would provide a more physiologically relevant model. Another limitation of our study is the restricted number and type of cell lines used. While we included two cell lines of different histotypes, this approach does not allow for broad conclusions regarding colon or gastric carcinoma. To provide more comprehensive insights, future studies should consider employing a panel of cell lines for each cancer type or, ideally, utilizing primary cells or organoids derived directly from these cancers.

## 5. Conclusions

Our findings indicate that Tempol exerts its anti-cancer activity through multifaceted and interconnected mechanisms. It primarily achieves this by inducing apoptosis and oxidative stress while concurrently suppressing pro-survival signaling pathways. Therefore, Tempol’s efficacy as an anti-cancer agent in these contexts appears to stem from its ability to generate damaging reactive oxygen species and also, critically, to deplete the cancer cells’ capacity to counteract this stress. This forces the cells past a crucial “redox tipping point,” where their defense mechanisms are exhausted and pro-death signals irrevocably dominate, ultimately leading to programmed cell death. These results underscore Tempol’s potential as a therapeutic agent for gastric and colon cancers.

## Figures and Tables

**Table 1:** Analyses of pro-apoptotic (Bax and cleaved caspase-3) proteins and anti-apoptotic (Bcl-2) proteins after Tempol treatment in CRL-1739 and HT29 cell lines.

	CRL-1739 Cell Line			HT29 Cell Line		
Proteins	Control (CRL-1739)	CRL-1739 + TPL	*p* value *, 95%CI (lower–upper)	Control (HT29)	HT29 + TPL	*p* value **, 95%CI (lower–upper)
Bax	0.85 ± 0.10 pg/mL	4.67 ± 1.03 pg/mL	<0.0001, [2.88–4.76]	0.967 ± 0.22 pg/mL	4.0 ± 0.65 pg/mL	<0.0001, [2.41–3.66]
Cleaved caspase-3	0.20 ± 0.12 pg/mL	5.17 ± 1.72 pg/mL	<0.0001, [3.40–6.54]	0.32 ± 0.11 pg/mL	4.17 ± 0.02 pg/mL	<0.0001, [2.78–4.92]
BCL-2	8.13 ± 0.83 pg/mL	3.75 ± 0.10 pg/mL	<0.0001, [−5.56–−3.21]	7.42 ± 1.56 pg/mL	3.00 ± 1.10 pg/mL	<0.0001, [−6.16–−2.70]

Notes: Results are presented as mean ± SD, *p* value *; comparison between CR-1739 and CRL-1739 plus TPL, *p* value **; comparison between HT29 and HT29 plus TPL, 95%CI represents the difference between the groups, Bax: Bcl-2-Associated X Protein, BCL-2: B-cell leukemia/lymphoma 2.

**Table 2:** Analyses of the levels of the phosphorylation levels of key signaling molecules after Tempol treatment in CRL-1739 and HT29 cell lines.

	CRL-1739 Cell Line			HT29 Cell Line		
Proteins	Control (CRL-1739)	CRL-1739 + TPL	*p* value *, 95%CI (lower–upper)	Control (HT29)	HT29 + TPL	*p* value **, 95%CI (lower–upper)
JNK	2.97 ± 0.28 pg/mL	1.30 ± 0.20 pg/mL	<0.0001, [−1.98–−1.35]	2.65 ± 0.23 pg/mL	1.23 ± 0.23 pg/mL	<0.0001, [−1.71–−1.13]
mTOR	26.17 ± 4.26 pg/mL	14.17 ± 1.47 pg/mL	<0.0001, [−16.10–7.90]	28.17 ± 4.44 pg/mL	12.83 ± 3.19 pg/mL	<0.0001, [−20.31–−10.36]
ERK	1.13 ± 0.14 pg/mL	0.44 ± 0.21 pg/mL	<0.0001, [−0.91–0.46]	1.27 ± 0.08 pg/mL	0.46 ± 0.14 pg/mL	<0.0001, [−1.15–−0.47]
AKT	9.83 ± 1.17 pg/mL	5.5 ± 0.84 pg/mL	<0.0001, [−5.64–3.02]	12 ± 1.67 pg/mL	6.67 ± 1.63 pg/mL	<0.0001, [−7.46–−3.20]

Notes: Results are presented as mean ± SD, *p* value *; comparison between CR-1739 and CRL-1739 plus TPL, *p* value **; comparison between HT29 and HT29 plus TPL, 95% CI represents the difference between the groups, ERK: Extracellular signal-Regulated Kinase, JNK: c-Jun N-terminal Kinase, AKT: Protein Kinase B, mTOR: mammalian Target of Rapamycin.

**Table 3:** Analyses of the Cellular Stress- and Apoptosis-Related Proteins after Tempol treatment in CRL-1739 and HT29 cell lines.

	CRL-1739 Cell Line			HT29 Cell Line		
Proteins	Control (CRL-1739)	CRL-1739 + TPL	*p* value *, 95%CI (lower–upper)	Control (HT29)	HT29 + TPL	*p* value **, 95%CI (lower–upper)
GRP78	0.35 ± 0.06 pg/mL	1.07 ± 0.13 pg/mL	<0.0001, [0.59–0.85]	0.56 ± 0.13 pg/mL	1.13 ± 0.17 pg/mL	<0.0001, [0.38–0.76]
AIF	0.35 ± 0.07 pg/mL	2.60 ± 0.41 pg/mL	<0.0001, [1.88–2.62]	0.43 ± 0.13 pg/mL	2.31 ± 0.60 pg/mL	<0.0001, [1.33–2.44]
GADD153	0.38 ± 0.17 pg/mL	1.25 ± 0.24 pg/mL	<0.0001, [0.61–1.14]	0.34 ± 0.09 pg/mL	1.08 ± 0.19 pg/mL	<0.0001, [0.55–0.9]
WEE1	0.33 ± 0.14 pg/mL	1.73 ± 0.24 pg/mL	<0.0001, [1.15–1.66]	0.37 ± 0.16 pg/mL	1.27 ± 0.25 pg/mL	<0.0001, [0.63–1.17]

Notes: Results are presented as mean ± SD, *p* value *; comparison between CR-1739 and CRL-1739 plus TPL, *p* value **; comparison between HT29 and HT29 plus TPL, 95%CI represents the difference between the groups, WEE1: Wee1-like protein kinase, GADD153: Growth Arrest and DNA Damage-inducible protein 153, GRP78: Glucose-Regulated Protein 78, AIF: Apoptosis-Inducing Factor.

**Table 4:** Analyses of the levels of Total Oxidant Status (TOS) and Total Antioxidant Status (TAS).

	CRL-1739 Cell Line			HT29 Cell Line		
Parameter	Control (CRL-1739)	CRL-1739 + TPL	*p* value *, 95%CI (lower–upper)	Control (HT29)	HT29 + TPL	*p* value **, 95%CI (lower–upper)
TOS (μmolH_2_O_2_ Eq./g)	12 ± 1.79	20.83 ± 2.71	<0.0001, [5.81–11.85]	10.17 ± 1.72	21.50 ± 2.88	<0.0001, [8.18–14.48]
TAS (mmol Trolox Eq./g)	1.75 ± 0.19	0.98 ± 0.15	<0.0001, [−0.99–−0.54]	2.10 ± 0.32	1.23 ± 0.16	<0.0001, [−1.21–−0.52]

Notes: Results are presented as mean ± SD, *p* value *; comparison between CR-1739 and CRL-1739 plus TPL, *p* value **; comparison between HT29 and HT29 plus TPL, 95%CI represents the difference between the groups.

## Data Availability

The data that support the findings of this study are available from the corresponding author upon reasonable request.

## Abbreviations

ROS	Reactive Oxygen Species
MAPK	Mitogen-Activated Protein Kinase
Akt/mTOR	Protein Kinase B/Mammalian Target of Rapamycin
ERK	Extracellular Signal-Regulated Kinase
Bax	Bcl-2-Associated X Protein
JNK	c-Jun N-Terminal Kinase AKT
AKT	Protein Kinase B
mTOR	Mammalian Target of Rapamycin
*p*-ERK	Phosphorylated Extracellular Signal-Regulated Kinase
*p*-JNK	Phosphorylated c-Jun N-terminal Kinase
*p*-AKT	Phosphorylated Protein Kinase B
*p*-mTOR	Phosphorylated Mammalian Target of Rapamycin
GADD153	Growth Arrest and DNA Damage-Inducible Protein 153
GRP78	Glucose-Regulated Protein 78
AIF	Apoptosis-Inducing Factor
PI3K	Phosphoinositide 3-Kinase
PTEN	Phosphatase and Tensin Homolog
KRAS	Kirsten Rat Sarcoma Viral Oncogene Homolog
BRAF	B-Raf Proto-Oncogene, Serine/Threonine Kinase
MEK	Mitogen-Activated Protein Kinase Kinase
HER2	Human Epidermal Growth Factor Receptor 2
ELISA	Enzyme-Linked Immunosorbent Assay
EGFR	Epidermal Growth Factor Receptor
GPCRs	G Protein-Coupled Receptors
VEGF	Vascular Endothelial Growth Factor
VEGFR	Vascular Endothelial Growth Factor Receptor
RAS	Rat Sarcoma Viral Oncogene
JNK1	c-Jun N-Terminal Kinase 1
p38-MAPK	p38 Mitogen-Activated Protein Kinase
